# The association between COVID-19 preventive behaviors and mental health conditions

**DOI:** 10.1371/journal.pone.0289533

**Published:** 2023-08-04

**Authors:** Elizabeth Wachira, Bhakti Chavan, Carolyn Nganga-Good, Caroline Kingori

**Affiliations:** 1 Department of Health and Human Performance, Texas A&M University- Commerce, Commerce, Texas, United States of America; 2 College of Osteopathic Medicine, Ohio University, Athens, Ohio, United States of America; 3 AWN Foundation, Baltimore, Maryland, United States of America; 4 College of Health Sciences and Professions, Ohio University, Athens, Ohio, United States of America; Atish Dipankar University of Science and Technology, BANGLADESH

## Abstract

**Background:**

An unintended consequence of COVID-19 quarantine preventive measures, is the increased prevalence of anxiety and depression. The purpose of this study was to examine the association between COVID-19 preventive behaviors and mental health conditions.

**Methods:**

A cross-sectional study was conducted using secondary data collected weekly from US adults aged 18 and older nationwide as part of the COVID-19 Household Impact Survey (CIS) from the University of Chicago. Logistic regression examined associations between COVID-19 preventive behaviors (wearing a face mask, washing or sanitizing hands, and keeping six-feet distance from those outside their household), mental health conditions (self-reporting feeling nervous, anxious, or on edge, feeling lonely, and feeling hopeless about the future and a history of a mental health condition) and demographic factors.

**Results:**

Majority of study participants were under 60 years (62.2%), female (55.8%), and non-Hispanic White (72.2%). Overall, participants more likely to have followed all three COVID-19 measures were those who reported high psychological distress compared to those with low distress for feeling anxious (adj. OR 1.16, 95% CI: 1.06–1.28, p = 0.002), lonely (adj. OR 1.12, 95% CI: 1.02–1.23, p = 0.019) or hopeless (adj. OR 1.10, 95% CI: 1.00–1.21, p = 0.043) for more than a day during the past 7 days.

**Conclusion:**

Our findings highlight that individuals with mental health conditions reported more psychological distress. Specifically, feeling depressed, anxious, lonely, and hopeless were triggered and exacerbated as a result of the pandemic and may have long-term effects on general well-being and productivity. Therefore, our findings have important implications on the need to include mental health promotion as part of pandemic response efforts. This includes developing policies and allocating funding so as to ensure sustainable mental health interventions and support, public and provider education on the importance of screening for mental health issues.

## Introduction

Since its emergence in early 2020, the novel coronavirus disease 2019 (COVID-19) has become the greatest global health crisis to date [1]. As of October 2022, approximately 624 million people have been infected with COVID-19 globally and over six million people have lost their lives due to the virus. In the United States, approximately ninte-six million people have been infected and one million lives have been lost [2]. Prior to vaccine availability, transmission prevention efforts focused on behavioral changes such as handwashing, social distancing and wearing facemasks [3, 4]. In addition, global quarantine and isolation measures were enforced to reduce community transmission through lockdowns and stay-at-home orders [5–7]. As a result, most public spaces were closed, social activities were limited and only essential services were operational, resulting in increased levels of loneliness, fear and psychological distress [6].

Psychological distress is an emotional response to an event perceived as threatening and is characterized by mental health conditions of anxiety, depression, and worry, which have progressively become psychological responses to COVID-19 [1, 5]. For example, looking at the impact of quarantine, a study conducted in China found that the prevalence of anxiety and depression was higher for individuals affected by quarantine compared to those unaffected in February 2020. For the affected, anxiety and depression rates were 12.9% and 22.4% respectively compared to those unaffected at 6.7% and 11.9% respectively [8].

Mental health conditions such as anxiety and depression can potentially affect one’s mental health, invoke irrational fear, often linked to work stress, alcohol abuse, suicidal ideations and more long term consequences tied to experiencing trauma [5, 9]. Where COVID-19 is concerned, fear and anxiety may have positively impacted preventive behaviors such that those with a fear of COVID-19, (coronaphobia), were likely to have an increase in positive attitude towards the vaccine [1].

Another related consequence of COVID-19 is the increase of health anxiety, which occurs when perceived body changes are interpreted as disease symptoms, and ranges from low to high anxiety [10]. For those with high health anxiety, maladaptive behavior similar to those experiencing coronaphobia include excessive hand washing, social withdrawal and panic purchasing, behaviors consistent with recommended public health mitigation efforts [10]. Low levels of health anxiety also impact health behaviors with these individuals perceiving themselves to be of low risk and less likely to follow recommended preventative practices such as handwashing [10].

A meta-analysis looking at the prevalence of depression during the COVID-19 pandemic found that globally, the pooled prevalence was 25% in 2020, a rate 7 times higher than in 2017 at 3.4% [11]. In the United States, mental health data collected before [June 2019] and during the pandemic (April 2020) show that the number of individuals with symptoms of anxiety increased from 8.2% to 30.8% and 6.6% to 23.5% for depression [12]. While COVID-19 psychological effects present across all populations, it differs based on various demographic factors. For example, studies show that those most likely to experience psychological distress are women, young adults, individuals with lower socioeconomic status, those living in rural and hard hit areas or have a history of chronic or mental health conditions [6, 8, 13, 14]. Furthermore, children and adolescents also experienced increases in various mental health conditions [12]. For example, a review of studies focused on the impact of COVID-19 on youth mental health cited studies reporting depression rates ranging from 22.6% to 43.7% and anxiety rates from 18.9% to 37.4% [15]. Such a steep increase in mental health conditions is alarming and measures need to be put in place to mitigate contributing factors.

The burden of COVID-19 coupled with the unintended consequences of pandemic response efforts, extends beyond physical health to include increases in various mental health conditions [6]. This highlights not only the psychological impact of COVID-19 to date, but the need to implement timely public health initiatives given that the mental health crisis is considered a “second pandemic” [12, 13]. The purpose of this study was to examine the association between COVID-19 preventive behaviors and mental health conditions in the US. The role of mental health in ensuring optimal health continues to grow in importance and is evidenced in its inclusion in the Sustainable Development Goals [24]. Therefore, this study will not only add to the literature on how the pandemic may have impacted mental health conditions, but can add insight into how this impact differed among various demographic groups and help inform targeted response interventions.

## Methods

This study used data from the COVID-19 Household Impact Survey (CIS) conducted by the nonpartisan and objective research organization (NORC) at the University of Chicago for the Data Foundation [16]. CIS is a cross-sectional, nationally representative household survey that collects estimates for preventative behaviors, physical and mental health, economic security, and other social dynamic factors during the COVID-19 pandemic. The survey provides weekly data estimates of US adults aged 18 and older nationwide and for 18 regional areas, including 10 states and 8 metropolitan statistical areas. This study uses CIS data from three time points in 2020: weeks 1–3, collected on April 20–26 (n = 2190), May 4–10 (n = 2288) and May 30 –June 8 (n = 2047), respectively [16]. This is de-identified publicly available data and therefore did not require IRB review. Detailed CIS study methods on recruitment and sampling are reported elsewhere [16].

### Measures

Our outcome variable, COVID-19 preventive behaviors, were assessed using participants’ responses to the following questions: *Which of the following measures*, *if any*, *are you taking in response to the coronavirus*? Our study focused on the three commonly recommended preventive behaviors: wearing a face mask; washing or sanitizing hands, and keeping six feet distance from those outside their household. Only those who selected ‘yes’ to practicing all three preventive behaviors were included in the study. Mental health conditions were based on two key questions. The first was a history of a mental health condition, based on a participant’s self-reported response (yes/no) to the following question: *Has a doctor or other health–care provider ever told you that you have a mental health condition*? We defined those who selected “yes” as individuals with a mental health condition and those who said “no” as not having a mental health condition.

The second measure of mental health conditions was psychological distress, based on a participant’s self-reported response to the following questions: *in the past 7 days*, *how often have you felt*: *nervous*, *anxious*, *or on edge*, *felt depressed*, *felt lonely*, *felt hopeless about the future*? Participant’s response options included four options: “not at all or less than 1 day, 1–2 days, 3–4 days, 5–7 days. We defined participants based on their selections and defined them as “not at all or less than a day” for those who selected this response and “more than one day” for those who selected all other options.

Quarantine impact was assessed using participants’ self-reported response (yes/no) to the following question specific to lockdown measures: “*In the past 7 days*, *have your personal plans been changed or affected by the following types of restrictions*, *or not*. We defined those who selected “yes” specifically to quarantine requirements or stay at home orders as being affected by quarantine and those who said “no” as not being affected.

### Covariates

The following covariates were included in the study analysis: age categories (18–29, 30–44, 45–59, 60+), gender (male, female), race/ethnicity categories (White, non-Hispanic, Black non-Hispanic, Hispanic, Other, non-Hispanic), household income (under $10,000, $10,000 –$30,000, $30,001 to $50,000, $50,001 to $100,001, over $100,001), education categories (no high school diploma, high school graduate or equivalent, some college, Bachelor’s degree or above), household size (one person,/live by self, two person, three persons, five persons, six or more persons), census region (northeast, midwest, south, west), population density determined based on 2010 US Census data (rural, suburban, urban), language (English, Spanish), and testing for COVID-19 infection using a q-tip to swab your cheek or nose if these options were available to you, how likely would you be to participate in them? (extremely likely, very likely, moderately likely, not too likely, not likely at all).

### Statistical analysis

Descriptive statistics was performed to summarize and describe the distribution of different variables. Using chi-square (*χ*^2^) test statistics, bivariate analyses were performed to compare study participants who used all COVID-19 related preventive behaviors and those who did not use all preventive behaviors by all the study variables. Logistic regression analysis was used to determine association between preventive behaviors use and study variables. The corresponding odds ratio (OR), 95% confidence interval (95% CI), and *p*-value were determined. Potential multicollinearity was assessed using variance inflation factor. Using a conservative cutoff threshold of VIF greater than or equal to 4, it was found that the regression analysis was not prone to multicollinearity. All analyses were conducted using SAS 9.4 (SAS Institute, Inc., Cary, NC). All *p*-values were two sided, and statistical significance was set as *p* < 0.05.

## Results

The final analytical sample size was 19,354. Majority of the study participants were under the age of 60 (62.2%), female (55.8%), non-Hispanic white (72.2%), had household income of $50k to under $100k (33.0%) and had a BA or above education (52.5%) lived in urban areas (80.4%), and spoke English (98.8%). Also, 0.6% reported to be told by a doctor or other health care provider that they had COVID-19, 0.7% reported to be told by a doctor or other health care provider that someone they lived with had COVID-19. 79.1% reported to undertake all three preventive behaviors (wear mask, use hand sanitizer, and maintain social distancing). 37.5% reported feeling nervous, anxious, or on edge for more than a day during the past 7 days. 38.2% reported feeling depressed for more than a day during the past 7 days. 37.9% reported feeling lonely for more than a day during the past 7 days. 38% reported feeling hopeless about the future for more than a day during the past 7 days. (Table 1).

**Table 1:** Overall characteristics of the study population and association with safety measures.

Study Population Characteristics		Overall	Safety measures		
			Not all three	All three	
		N (%)	N (%)	N (%)	p value
**Age**					<0.001***
	18–29	2507 (13.0)	763 (30.4)	1744 (69.6)	
	30–44	4923 (25.4)	1213 (24.6)	3710 (75.4)	
	45–59	4626 (23.9)	919 (19.9)	3707 (80.1)	
	60+	7298 (37.7)	1151 (15.8)	6147 (84.2)	
**Gender**					<0.001***
	Female	10803 (55.8)	1950 (18.1)	8853 (82.0)	
	Male	8551 (44.2)	2096 (24.5)	6455 (75.5)	
**Race-ethnicity**					0.138
	non-Hispanic White	13981 (72.2)	2942 (21.0)	11039 (79.0)	
	non-Hispanic Black	1905 (9.8)	361 (19.0)	1544 (81.1)	
	Hispanic	1940 (10.0)	423 (21.8)	1517 (78.2)	
	Other	1528 (7.9)	320 (20.9)	1208 (79.1)	
**Household income**					<0.001***
	Under $10,000	957 (4.9)	281 (29.4)	676 (70.6)	
	$10,000 to under $30,000	3170 (16.4)	845 (26.7)	2325 (73.3)	
	$30,000 to under $50,000	3265 (16.9)	736 (22.5)	2529 (77.5)	
	$50,000 to under $100,000	6386 (33.0)	1318 (20.6)	5068 (79.4)	
	$100,000 or more	5576 (28.8)	866 (15.5)	4710 (84.5)	
**Education**					<0.001***
	No HS diploma	655 (3.4)	192 (29.3)	463 (70.7)	
	HS graduate or equivalent	2495 (12.9)	726 (29.1)	1769 (70.9)	
	Some college	6042 (31.2)	1467 (24.3)	4575 (75.7)	
	BA or above	10162 (52.5)	1661 (16.4)	8501 (83.7)	
**Household size**					<0.001***
	One person, I live by myself	5805 (30.0)	1267 (21.8)	4538 (78.2)	
	Two persons	6656 (34.4)	1211 (18.2)	5445 (81.8)	
	Three persons	2779 (14.4)	547 (19.7)	2232 (80.3)	
	Four persons	2078 (10.7)	490 (23.6)	1588 (76.4)	
	Five persons	1026 (5.3)	224 (21.8)	802 (78.2)	
	Six or more persons	1010 (5.2)	307 (30.4)	703 (69.6)	
**Region**					<0.001***
	Northeast	2616 (13.5)	361 (13.8)	2255 (86.2)	
	Midwest	5381 (27.8)	1295 (24.1)	4086 (75.9)	
	South	7013 (36.2)	1496 (21.3)	5517 (78.7)	
	West	4344 (22.4)	894 (20.6)	3450 (79.4)	
**Population density**					<0.001***
	Rural	924 (4.8)	296 (32.0)	628 (68.0)	
	Suburban	2866 (14.8)	758 (26.5)	2108 (73.6)	
	Urban	15564 (80.4)	2992 (19.2)	12572 (80.8)	
**Language**					0.025*
	English	19115 (98.8)	4010 (21.0)	15105 (79.0)	
	Spanish	239 (1.2)	36 (15.1)	203 (84.9)	
**Likelihood to test for COVID-19 if options were available**					<0.001***
	Extremely likely	6241 (32.3)	788 (12.6)	5453 (87.4)	
	Very likely	4576 (23.6)	691 (15.1)	3885 (84.9)	
	Moderately likely	4223 (21.8)	923 (21.9)	3300 (78.1)	
	Not too likely	1906 (9.9)	590 (31.0)	1316 (69.1)	
	Not likely at all	2408 (12.4)	1054 (43.8)	1354 (56.2)	
**History of a Mental Health condition**					0.030*
	Yes	3242 (16.8)	632 (19.5)	2610 (80.5)	
	No	16112 (83.3)	3414 (21.2)	12698 (78.8)	
**Frequency of feeling nervous, anxious, or on edge**					<0.001***
	Not at all or less than 1 day	12095 (62.5)	2693 (22.3)	9402 (77.7)	
	More than a day	7259 (37.5)	1353 (18.6)	5906 (81.4)	
**Frequency of feeling depressed**					<0.001***
	Not at all or less than 1 day	11953 (61.8)	2636 (22.1)	9317 (78.0)	
	More than a day	7401 (38.2)	1410 (19.1)	5991 (81.0)	
**Frequency of feeling lonely**					<0.001***
	Not at all or less than 1 day	12012 (62.1)	2678 (22.3)	9334 (77.7)	
	More than a day	7342 (37.9)	1368 (18.6)	5974 (81.4)	
**Frequency of feeling hopeless about the future**					<0.001***
	Not at all or less than 1 day	11995 (62.0)	2669 (22.3)	9326 (77.8)	
	More than a day	7359 (38.0)	1377 (18.7)	5982 (81.3)	
**Changed plans due to quarantine/stay at home orders**					0.764
	No	12719 (65.7)	2667 (21.0)	10052 (79.0)	
	Yes	6635 (34.3)	1379 (20.8)	5256 (79.2)	
**Safety measures**					
	Not all three	4046 (20.9)			
	All three	15308 (79.1)			

N Frequency, % percentage

*p<0.05

***p<0.001

Bivariate analysis found a significant association between preventive behaviors and age, gender, household income, education, household size, region, population density, language, perform COVID test, had a clinically diagnosed mental health condition, and experiencing feelings of nervousness, anxiety, depression, loneliness, and hopelessness during the past 7 days (p < 0.05) (Table 1).

Table 2 looks at the association between clinically diagnosed mental health conditions and feelings of anxiety, depression, loneliness, and hopelessness. Among study participants with clinically diagnosed mental health conditions, a greater proportion reported experiencing all four psychological distress measures (anxious, depressed, lonely and hopeless) for more than a day during the past 7 days.

**Table 2:** Association between mental health conditions, quarantine requirements, and feelings of anxiety, depression, loneliness, hopelessness.

	History of a Mental Health condition			Changed plans due to quarantine/stay at home orders		
	No	Yes		No	Yes	
	N (%)	N (%)	p value	N (%)	N (%)	p value
**Frequency of feeling nervous, anxious, or on edge**			<0.001***			0.032*
Not at all or less than 1 day	11003 (91.0)	1092 (9.0)		8017 (66.3)	4078 (33.7)	
More than a day	5109 (70.4)	2150 (29.6)		4702 (64.8)	2557 (35.2)	
**Frequency of feeling depressed**			<0.001***			0.005**
Not at all or less than 1 day	10860 (90.9)	1093 (9.1)		7945 (66.5)	4008 (33.5)	
More than a day	5252 (71.0)	2149 (29.0)		4774 (64.5)	2627 (35.5)	
**Frequency of feeling lonely**			<0.001***			0.004**
Not at all or less than 1 day	10899 (90.7)	1113 (9.3)		7986 (66.5)	4026 (33.5)	
More than a day	5213 (71.0)	2129 (29.0)		4733 (64.5)	2609 (35.5)	
**Frequency of feeling hopeless about the future**			<0.001***			<0.001***
Not at all or less than 1 day	10890 (90.8)	1105 (9.2)		7996 (66.7)	3999 (33.3)	
More than a day	5222 (71.0)	2137 (29.0)		4723 (64.2)	2636 (35.8)	

N Frequency, % percentage

*p<0.05

**p<0.01

***p<0.001

Table 2 also looks at the association between personal plans being changed or affected by quarantine requirements or stay at home orders in the past 7 days and feelings of anxiety, depression, loneliness, and hopelessness. Among study participants whose personal plans changed or were affected by quarantine requirements or stay at home orders in the past 7 days, a greater proportion reported experiencing all four psychological distress measures (anxious, depressed, lonely and hopeless) for more than a day during the past 7 days.

Table 3 shows multivariate logistic regression analysis results. After adjusting for potential confounders, compared to study participants age 60+ years, all other age groups were less likely to follow all three preventive behaviors: 18–29 years (Adj. OR, 95% CI, p value) (0.35, 0.31–0.40, p < 0.001), 30–44 years (0.47, 0.42–0.52, p < 0.001), 45–59 years (0.64, 0.57–0.71, p < 0.001). After adjusting for potential confounders, sociodemographic differences in preventive behaviors were noted. Compared to males, females were more likely to follow all three preventive behaviors (1.56, 1.44–1.68, p<0.001), compared to non-Hispanic white, all other race/ethnicities were more likely to follow all three preventive behaviors: non-Hispanic black (1.53, 1.33–1.76, p<0.001), Hispanic (1.29, 1.12–1.48, p < 0.001), Other (1.18, 1.02–1.35, p = 0.022).

**Table 3:** Multivariate adjusted logistic regression analysis.

	All three safety measures	
	adj. OR (95% CI)	p value
**Age**		
60+	Reference	
18–29	0.35 (0.31–0.40)	<0.001***
30–44	0.47 (0.42–0.52)	<0.001***
45–59	0.64 (0.57–0.71)	<0.001***
**Gender**		
Male	Reference	
Female	1.56 (1.44–1.68)	<0.001***
**Race-ethnicity**		
non-Hispanic White	Reference	
non-Hispanic Black	1.53 (1.33–1.76)	<0.001***
Hispanic	1.29 (1.12–1.48)	<0.001***
Other	1.18 (1.02–1.35)	0.022*
**Household income**		
$100,000 or more	Reference	
Under $10,000	0.64 (0.53–0.77)	<0.001***
$10,000 to under $30,000	0.66 (0.58–0.75)	<0.001***
$30,000 to under $50,000	0.80 (0.71–0.91)	<0.001***
$50,000 to under $100,000	0.82 (0.74–0.91)	<0.001***
**Education**		
BA or above	Reference	
No HS diploma	0.71 (0.58–0.87)	0.001**
HS graduate or equivalent	0.62 (0.55–0.70)	<0.001***
Some college	0.73 (0.67–0.80)	<0.001***
**Household size**		
One person, I live by myself	Reference	
Two persons	1.19 (1.08–1.31)	<0.001***
Three persons	1.22 (1.08–1.38)	0.002**
Four persons	1.03 (0.90–1.18)	0.674
Five persons	1.29 (1.08–1.54)	0.005**
Six or more persons	0.94 (0.80–1.11)	0.495
**Region**		
South	Reference	
Northeast	1.76 (1.54–2.01)	<0.001***
Midwest	0.83 (0.76–0.91)	<0.001***
West	1.04 (0.94–1.15)	0.489
**Population density**		
Urban	Reference	
Rural	0.68 (0.58–0.80)	<0.001***
Suburban	0.79 (0.72–0.88)	<0.001***
**Language**		
English	Reference	
Spanish	1.71 (1.16–2.53)	0.008**
**Likelihood to test for COVID-19 if options were available**		
Not likely at all	Reference	
Extremely likely	4.36 (3.90–4.91)	<0.001***
Very likely	3.75 (3.33–4.23)	<0.001***
Moderately likely	2.53 (2.26–2.84)	<0.001***
Not too likely	1.65 (1.44–1.88)	<0.001***
**History of a Mental Health condition**		
No	Reference	
Yes	1.09 (0.97–1.21)	0.138
**Frequency of feeling nervous, anxious, or on edge**		
Not at all or less than 1 day	Reference	
More than a day	1.16 (1.06–1.28)	0.002**
**Frequency of feeling depressed**		
Not at all or less than 1 day	Reference	
More than a day	1.05 (0.95–1.15)	0.367
**Frequency of feeling lonely**		
Not at all or less than 1 day	Reference	
More than a day	1.12 (1.02–1.23)	0.019*
**Frequency of feeling hopeless**		
Not at all or less than 1 day	Reference	
More than a day	1.10 (1.00–1.21)	0.043*
**Changed plans due to quarantine/stay at home orders**		
Yes		
No	1.00 (0.92–1.08)	0.954

adj. OR adjusted odds ratio, 95% CI—95% confidence interval

*p<0.05

**p<0.01

***p<0001

In addition, compared to study participants with household income of $100k and more, all other income category groups were less likely to follow all three preventive behaviors: under 10k (0.64, 0.53–0.77, p < 0.001), 10k-30k (0.66, 0.58–0.75, p < 0.001), 30k-50k (0.80, 0.71–0.91, p < 0.001), 50k-100k (0.82, 0.74–0.91, p < 0.001). Compared to study participants with BA degree or above, all other educational groups were less likely to follow all three preventive behaviors: no HS diploma (0.71, 0.58–0.87, p = 0.001), HS graduate or equivalent (0.62, 0.55–0.70, p < 0.001), some college (0.73, 0.67–0.80, p < 0.001).

Compared to study participants with one person household size, those living in a two, three and five-person household were more likely to follow all three preventive behaviors: two persons (1.19, 1.08–1.31, p < 0.001), three persons (1.22, 1.08–1.38, p = 0.002), five persons (1.29, 1.08–1.54, p = 0.005). Compared to study participants living in the South, those living in Northeastern region were more likely to follow all three preventive behaviors (1.76, 1.54–2.01, p < 0.001), whereas those living in Midwestern region were less likely to follow all three preventive behaviors (0.83, 0.76–0.91, p < 0.001). Compared to study participants residing in urban areas, those living in rural and suburban areas were less likely to follow all three preventive behaviors: rural (0.68, 0.58–0.80, p < 0.001), suburban (0.79, 0.72–0.88, p <0.001). Compared to English speaking study participants, Spanish speakers were more likely to follow all three preventive behaviors (1.71, 1.16–2.53, p = 0.008).

Compared to study participants who were not likely at all to get tested for COVID-19, all other study participants were more likely to follow all three preventive behaviors: extremely likely (4.36,3.90–4.91, p < 0.001), very likely (3.75, 3.33–4.23, p < 0.001), moderately likely (2.53, 2.26–2.84, p < 0.001), not too likely (1.65, 1.44–1.88, p < 0.001).

Compared to study participants who did not feel nervous, anxious or on edge at all or for less than a day, those who reported feeling nervous, anxious or on edge for more than a day during the past 7 days were more likely to follow all three preventive behaviors (1.16, 1.06–1.28, p = 0.002). Compared to study participants who were not lonely at all or for less than a day, those who reported feeling lonely for more than a day during the past 7 days were more likely to follow all three preventive behaviors (1.12, 1.02–1.23, p = 0.019). And compared to study participants who did not feel hopeless, those who reported feeling hopeless for more than a day during the past 7 days were more likely to follow all three preventive behaviors (1.10, 1.00–1.21, p = 0.043).

## Discussion

The current study examined the association between COVID-19 preventive behaviors and mental health conditions in a nationally representative household survey. Age, gender, race, socioeconomic status, population density, region, language, and mental health conditions, predicted the likelihood of engaging in the three preventive behaviors (mask wearing, handwashing, social distancing) in the study.

In general, the majority of participants practiced all three preventive behaviors that are deemed effective in slowing down the spread of the infectious disease, disease burden and mortality [17, 18]. A closer assessment of the preventive behaviors across demographic groups revealed that participants who were 60 years and above were more likely to engage in the three behaviors compared to all other age groups. Duan et al. [19] also found that older adults were likely to engage in those three preventive behaviors based on various factors. For example, mask wearing and social distancing were influenced by health knowledge, past behavior, attitude, and subjective norms, while hand washing was influenced by how much control someone had on that action. On the contrary, Alivernini et al. [20] found that adolescents and young adults’ likelihood to engage in preventive behaviors such as physical distancing, was a factor of autonomous motivation. To effectively motivate adolescents and young adults to engage in the targeted behaviors, messaging and interventions should focus on emphasizing personal and social value of engaging in the behaviors.

Globally, the COVID-19 pandemic may have triggered and exacerbated mental health issues. For example, depression rates increased exponentially from 3.4% (2017) to 25% (2020) [11]. In the current study, less than 50% of the participants felt depressed, lonely, hopeless about the future and anxious or on edge for more than a day during the past seven days. However, mental health conditions differed significantly among those who had a clinically diagnosed mental health condition compared to those who did not, whereby those clinically diagnosed reported feeling more depressed, anxious, lonely and hopeless. Overall, heightened mental distress could be attributed to fear of the unknown, information overload, misinformation, isolation, and mortality concerns [21–23].

The role of mental health in ensuring optimal health continues to grow in importance and is evidenced in its inclusion in the Sustainable Development Goals [24]. While mental health conditions are common globally, those affected live one out of five years with a disability and it accounts for economic loss of over $1 trillion annually. Furthermore, elderly women and young people in resource limited countries are disproportionately impacted [25]. Between 1990 and 2019, mental health conditions accounted for an increase (3.1% to 4.9%) in the proportion of disability-adjusted life-years (DALYS). In the US, the prevalence and impact of mental health conditions follows a similar global trend whereby 1 in 5 Americans experience mental illness in a given year, and more than 50% of the population are expected to be diagnosed with a mental disorder at some point in their life [26]. Therefore, timely and sustained interventions are necessary to curtail the pervasive nature of mental health conditions.

Related to the current study, the World Health Organization recently examined the impact of lockdown restrictions on mental health. In 2020 findings revealed an increase in depression and anxiety globally by 25% and an increased number of people suffering from mental health issues [27]. Similarly, in the US, 1 in 5 adults reported that the pandemic had a significant impact on their mental health with the proportion of adults reporting symptoms of anxiety and depression increasing from 36.4 to 41.5% [14, 28]. In the current study, we examined the extent to which personal plans affected by travel restrictions were associated with mental conditions. For those participants whose personal plans were affected by travel restrictions in the past 7 days, most of them reported feeling hopeless, depressed, lonely, nervous, anxious or on edge. More research is needed to establish the causal pathways between COVID-19 and mental health conditions so as to create targeted interventions that are contextually relevant.

Evidence clearly shows that COVID-19 exacerbated mental health conditions yet investments in addressing them did not increase. Prior to the pandemic, inequalities in the allocation of funding to mental health compared to physical healthcare existed [29, 30]. During the pandemic, a 2020 World Health Organization rapid assessment found that among 25% of countries included in the assessment, at least 75% of mental health services were completely or partially disrupted. Therefore, the increase in the prevalence of mental health conditions add to an already strained healthcare system across the globe. Initiatives to ensure access for all, particular those at high risk inclusive of individuals with pre-existing mental health conditions are warranted [29]. Recommendations include capacity building, scaling up telemedicine access and integrating mental health services into settings such as primary care, education and community service. Doing so ensures that countries can meet the increased demand for adequate and accessible mental health and psychosocial support for all [30, 31].

While this cross-sectional study had representation from a nationally representative sample, the findings may not be generalizable to all populations and settings. Those who did not respond to the survey and those in different settings such as those in underserved and rural areas that may not have access to taking these kinds of surveys, may have had different experiences from those who responded to the survey. Self-reported results also have the likelihood of response bias where the participants may not have provided the most accurate data on their mental well-being and the COVID-19 preventative practices they practiced. Nevertheless, study findings and other similar studies, provide meaningful baseline evidence that can be explored further to develop sustainable efforts to screen for and address the long-term effects of the pandemic on mental well-being. Future research should further explore the association between the long-term effects of the COVID-19 pandemic on mental health status and adherence to preventive practices that can be utilized to address current and future public health crises. Future research should also further explore these associations in various demographic groups, settings, and persons with varying levels of health conditions to develop targeted education and interventions. Our findings have implications for mental health and public health education, policy, and practice efforts. It is imperative to educate healthcare providers as well as non-medical staff about the importance of screening for mental health and providing education and emotional support to all their patients regardless of their demographics and at various healthcare entry points. It is also imperative to develop policies and allocate funding as part of pandemic response efforts that support sustainable mental health interventions and support, as well as public and provider education.

## Conclusion

In conclusion, this study highlights the impact the COVID-19 pandemic may have had on mental health and the adherence of preventative practices for different demographic groups from a nationally representative sample in the USA. Mental health conditions such as feeling depressed, anxious, lonely, and hopeless were triggered and exacerbated as a result of pandemic response efforts and may have long-term effects on the general well-being and productivity. As such, future pandemic response efforts must plan for the impact and increase in demand for mental health services through integration of care and support into the primary healthcare and community setting.

## Supporting information

S1 File Study dataset. (XLSX)

## References

[1] Turan GB, Aksoy M, Özer Z, Demir C. The association between coronaphobia and attitude towards COVID-19 Vaccine: A sample in the east of Turkey. L’Encéphale. 2022 Feb; 48(1):38–42. doi: 10.1016/j.encep.2021.04.002 34243957 PMC8196324

[2] World Health Organization. WHO Coronavirus (COVID-19) Dashboard [Internet]. [cited 2022 Nov 21]. Available from: https://covid19.who.int

[3] Nguyen NPT, Hoang TD, Tran VT, Vu CT, Fodjo JNS, Colebunders R, et al. Preventive behavior of Vietnamese people in response to the COVID-19 pandemic. PLOS ONE. 2020 Sep 9; 15(9):e0238830. doi: 10.1371/journal.pone.0238830 32903286 PMC7480837

[4] Camacho-Rivera M, Islam JY, Rivera A, Vidot DC. Attitudes toward Using COVID-19 mHealth Tools among adults with chronic health conditions: Secondary data analysis of the COVID-19 Impact Survey. JMIR MHealth UHealth. 2020 Dec 17; 8(12):e24693. doi: 10.2196/24693 33301415 PMC7748389

[5] Olapegba PO, Chovwen CO, Ayandele O, Ramos-Vera C. Fear of COVID-19 and preventive health behavior: Mediating role of post-traumatic stress symptomology and psychological distress. Int J Ment Health Addict. 2022 Oct;20(5):2922–33. doi: 10.1007/s11469-021-00557-4 34121960 PMC8183581

[6] Pedrosa AL, Bitencourt L, Fróes ACF, Cazumbá MLB, Campos RGB, de Brito SBCS, et al. Emotional, behavioral, and psychological impact of the COVID-19 pandemic. Front Psychol. 2020 Oct 2; 11:566212. doi: 10.3389/fpsyg.2020.566212 33117234 PMC7561666

[7] Hossain MM, Sultana A, Purohit N. Mental health outcomes of quarantine and isolation for infection prevention: A systematic umbrella review of the global evidence. Epidemiol Health. 2020; 42. doi: 10.4178/epih.e2020038 32512661 PMC7644933

[8] Lei L, Huang X, Zhang S, Yang J, Yang L, Xu M. Comparison of prevalence and associated factors of anxiety and depression among people affected by versus people unaffected by quarantine during the COVID-19 Epidemic in Southwestern China. Med Sci Monit [Internet]. 2020 Apr 20 [cited 2022 Nov 21]; 26. Available from: https://www.medscimonit.com/abstract/index/idArt/924609 doi: 10.12659/MSM.924609 32335579 PMC7199435

[9] Acharibasam JW, Chireh B, Menegesha HG. Assessing anxiety, depression and insomnia symptoms among Ebola survivors in Africa: A meta-analysis. Schieffelin J, editor. PLOS ONE. 2021 Feb 5; 16(2):e0246515. doi: 10.1371/journal.pone.0246515 33544772 PMC7864444

[10] Asmundson GJG, Taylor S. How health anxiety influences responses to viral outbreaks like COVID-19: What all decision-makers, health authorities, and health care professionals need to know. J Anxiety Disord. 2020 Apr; 71:102211. doi: 10.1016/j.janxdis.2020.102211 32179380 PMC7271220

[11] Bueno-Notivol J, Gracia-García P, Olaya B, Lasheras I, López-Antón R, Santabárbara J. Prevalence of depression during the COVID-19 outbreak: A meta-analysis of community-based studies. Int J Clin Health Psychol. 2021 Jan; 21(1):100196. doi: 10.1016/j.ijchp.2020.07.007 32904715 PMC7458054

[12] Lamar MR, Speciale M, Forbes LK, Donovan C. The mental health of U.S. parents during the COVID-19 Pandemic. J Ment Health Couns. 2021 Oct 1; 43(4):319–35.

[13] Choi KR, Heilemann MV, Fauer A, Mead M. A second pandemic: Mental health spillover from the novel coronavirus (COVID-19). J Am Psychiatr Nurses Assoc. 2020 Jul; 26(4):340–3. doi: 10.1177/1078390320919803 32340586

[14] National Alliance of Mental Illness. Mental health by the Numbers [Internet]. About mental health. [cited 2022 Nov 21]. Available from: https://www.nami.org/mhstats

[15] Nearchou F, Flinn C, Niland R, Subramaniam SS, Hennessy E. Exploring the impact of COVID-19 on mental health outcomes in children and adolescents: A systematic review. Int J Environ Res Public Health. 2020 Nov 16; 17(22):8479. doi: 10.3390/ijerph17228479 33207689 PMC7698263

[16] Wozniak W. COVID Impact Survey: Version 1–3 [Dataset]. Chicago, IL: National Opinion Research Center. Available from: https://www.covid-impact.org/results

[17] Pan L, Wang J, Wang X, Ji JS, Ye D, Shen J, et al. Prevention and control of coronavirus disease 2019 (COVID-19) in public places. Environ Pollut. 2022 Jan 1; 292:118273. doi: 10.1016/j.envpol.2021.118273 34634404 PMC8498926

[18] Sharif N, Alzahrani KJ, Ahmed SN, Opu RR, Ahmed N, Talukder A, et al. Protective measures are associated with the reduction of transmission of COVID-19 in Bangladesh: A nationwide cross-sectional study. PLOS ONE. 2021 Nov 22; 16(11):e0260287. doi: 10.1371/journal.pone.0260287 34807962 PMC8608304

[19] Duan Y, Shang B, Liang W, Lin Z, Hu C, Baker JS, et al. Predicting hand washing, mask wearing and social distancing behaviors among older adults during the covid-19 pandemic: an integrated social cognition model. BMC Geriatr. 2022; 22(1):1–16. 35109798 10.1186/s12877-022-02785-2 PMC8807958

[20] Alivernini F, Manganelli S, Girelli L, Cozzolino M, Lucidi F, Cavicchiolo E. Physical distancing behavior: The role of emotions, personality, motivations, and moral decision-making. J Pediatr Psychol. 2021; 46(1):15–26. doi: 10.1093/jpepsy/jsaa122 33355343 PMC7798981

[21] World Health Organization. Countering misinformation about COVID-19 [Internet]. [cited 2022 Nov 21]. Available from: https://www.who.int/news-room/feature-stories/detail/countering-misinformation-about-covid-19

[22] Cramer H. The other pandemic: Mental health before, during, and after COVID-19. Vol. 28, Journal of Integrative and Complementary Medicine. Mary Ann Liebert, Inc., publishers 140 Huguenot Street, 3rd Floor New …; 2022. p. 108–9. 35108127 10.1089/jicm.2022.0473

[23] Qian M, Wu Q, Wu P, Hou Z, Liang Y, Cowling BJ, et al. Psychological responses, behavioral changes and public perceptions during the early phase of the COVID-19 outbreak in China: a population based cross-sectional survey. MedRxiv. 2020; 10.1136/bmjopen-2020-040910 PMC7545627 33033099

[24] World Health Organization. Mental health [Internet]. [cited 2022 Nov 21]. Available from: https://www.who.int/health-topics/mental-health#tab=tab_1

[25] World Health Organization. The WHO special initiative for mental health (2019–2023): universal health coverage for mental health [Internet]. World Health Organization; 2019. Available from: https://apps.who.int/iris/handle/10665/310981

[26] Center for Disease Control and Prevention. About Mental Health [Internet]. 2021 [cited 2022 Nov 21]. Available from: https://www.cdc.gov/mentalhealth/learn/index.htm

[27] Freeman M. The World Mental Health Report: transforming mental health for all. World Psychiatry. 2022; 21(3):391. doi: 10.1002/wps.21018 36073688 PMC9453907

[28] Vahratian A, Blumberg SJ, Terlizzi EP, Schiller JS. Symptoms of anxiety or depressive disorder and use of mental health care among adults during the COVID-19 pandemic—United States, August 2020–February 2021. Morb Mortal Wkly Rep. 2021; 70(13):490. 10.15585/mmwr.mm7013e2 PMC8022876 33793459

[29] McCartan C, Adell T, Cameron J, Davidson G, Knifton L, McDaid S, et al. A scoping review of international policy responses to mental health recovery during the COVID-19 pandemic. Health Research Policy and Systems. 2021 Apr 6;19(1):58. doi: 10.1186/s12961-020-00652-3 33823855 PMC8022299

[30] Maulik PK, Thornicroft G, Saxena S. Roadmap to strengthen global mental health systems to tackle the impact of the COVID-19 pandemic. Int J Ment Health Syst. 2020 Jul 29;14(1):57. doi: 10.1186/s13033-020-00393-4 32742305 PMC7389161

[31] Tausch A, e Souza RO, Viciana CM, Cayetano C, Barbosa J, Hennis AJ. Strengthening mental health responses to COVID-19 in the Americas: A health policy analysis and recommendations. The Lancet Regional Health—Americas. 2022 Jan 1;5:100118. doi: 10.1016/j.lana.2021.100118 35098200 PMC8782269

